# Implementation Science Competencies for Policy Transformation Framework (ISCPT)

**DOI:** 10.3390/healthcare13070723

**Published:** 2025-03-25

**Authors:** Modi Al-Moteri, Jamil Aljuaid, Hayat Mohammed Alqurashi, Mashael Mohammed Otayni, Muneera Hasheem Al-Jaid, Amira Mohamed Hamed Ahmed, Bandar Obaid Al Sufyani, Saeed Atiah Almalki, Anare Dinnesse Cagoco, Rana Mohammed Bamansur, Digna Fatalla, Shara Hamad Muqree, Atheer Mutair Ammar Alkhaldi, Fatemah Nooralhak Turdi, Maaidah M. Algamdi, Rizal Angelo N. Grande, Daniel Joseph E. Berdida, Alalyani Mesheil, Emad Althobaiti

**Affiliations:** 1 Medical Surgical Nursing Department, College of Nursing, Taif University, P.O. Box 11099, Taif 21944, Saudi Arabia; 2 Children’s Hospital, Taif Health Cluster, Ministry of Health, Taif 21944, Saudi Arabia; jaaljuaid@moh.gov.sa (J.A.); hamalgurashi@moh.gov.sa (H.M.A.); motaini@moh.gov.sa (M.M.O.); moteby@moh.gov.sa (M.H.A.-J.); amiramh@moh.gov.sa (A.M.H.A.); bal-sofyani@moh.gov.sa (B.O.A.S.); salmalki34@moh.gov.sa (S.A.A.); acagoco@moh.gov.sa (A.D.C.); rbamansur@moh.gov.sa (R.M.B.); dfatalla@moh.gov.sa (D.F.); smqree@moh.gov.sa (S.H.M.); atalkhaldi@moh.gov.sa (A.M.A.A.); fturdi@moh.gov.sa (F.N.T.); 3 Community and Mental Health Nursing Department, Faculty of Nursing, University of Tabuk, Tabuk 47512, Saudi Arabia; ialghamdi@ut.edu.sa; 4 Department of Nursing, Fakeeh College for Medical Sciences, Jeddah 23323, Saudi Arabia; rgrande@fcms.edu.sa; 5 Department of Nursing, North Private College of Nursing, Arar 73215, Saudi Arabia; daniel@nec.edu.sa; 6 Nursing College, Khamis Mushait, King Khalid University, Abha 62521, Saudi Arabia; malalyani@kku.edu.sa; 7 King Abdulaziz Medical City, Ministry of National Guard Health Affairs, Jeddah 22384, Saudi Arabia; althobaitiem@mngha.med.sa

**Keywords:** nursing, competencies, implementation science, EBP

## Abstract

Implementation science (IS) models play a crucial role in translating evidence-based practice (EBP) into sustainable policy reforms. However, the competencies required for nurses to lead these transformations remain poorly defined. **Objective**: This study develops a framework for implementation lead (IL) nurses, identifying the core competencies needed to drive evidence-based policy transformation within healthcare systems. **Method:** A secondary data analysis (SDA) was conducted using qualitative data from focus group interviews originally collected, recorded, and transcribed as part of the EQUIP (Evidence-based Quality Improvement Project). The dataset includes insights from 12 IL nurses who participated in PEACE-based training, addressing real-world clinical challenges. Their perspectives were thematically analyzed to generate a competency framework for policy leadership. **Findings:** The study developed the Implementation Science Competencies for Policy Transformation (ISCPT) framework, which highlights three pillars: (1) evidence appraisal and guideline development, (2) collaborative leadership for policy advocacy, and (3) continuous improvement through data-driven decision-making. **Conclusions**: Grounded in IL nurses’ perspectives, the ISCPT framework provides a nurse-centric roadmap for policy transformation, integrating interdisciplinary collaboration, adaptive leadership, and evidence-based decision-making into nursing education and practice. While the findings reflect a single healthcare context, the framework offers actionable guidance for preparing nurses to lead policy-driven healthcare improvements.

## 1. Introduction

Healthcare systems worldwide are undergoing transformative changes to address growing complexities [1]. Nurses are crucial in these reforms, significantly impacting patient care and health outcomes [2]. Implementation lead (IL) nurses have emerged as key agents of change [3]. Grounded in implementation science (IS), IL nurses—sometimes referred to as nurse scientists—help bridge the gap between research and practice, ensuring the effective integration of evidence-based practices (EBPs) into routine care [4].

IS is a rapidly evolving field dedicated to bridging the gap between research and practice, ensuring that evidence-based interventions are effectively integrated into real-world healthcare settings [5]. IS not only examines how best to apply, adapt, and sustain these innovations within complex healthcare systems but also addresses key challenges such as organizational resistance, resource limitations, and behavioral factors that impact change adoption. By leveraging implementation frameworks and models, IS provides structured methodologies to assess barriers to change, optimize workflow integration, and evaluate the long-term sustainability of evidence-based interventions [6].

A critical aspect of implementation science is its emphasis on context-specific adaptation, recognizing that interventions that are successful in one setting may require modifications to be effective in another. It promotes stakeholder engagement, interdisciplinary collaboration, and continuous feedback loops to ensure that healthcare innovations are not only adopted but also sustained over time. This is particularly relevant to IL nurses, who are pivotal in driving practice transformations, advocating for evidence-driven policies and ensuring that clinical guidelines evolve in response to emerging challenges.

The PEACE framework—problem identification, evidence review, appraisal of evidence, changing practice, and evaluation—provides IL nurses with a structured approach to integrating EBPs [7]. By systematically identifying gaps, assessing research relevance, and facilitating sustainable change, IL nurses can navigate institutional barriers, enhance compliance with best practices, and improve patient outcomes. As healthcare systems rapidly evolve, implementation science ensures that research is effectively embedded into everyday practice, keeping policies dynamic, evidence-driven, and responsive to emerging healthcare needs [8].

The significance of IL nurses lies in their ability to drive change and transformation at all levels of the healthcare system. Unlike traditional leadership roles tied to formal authority, IL nurses lead through influence, collaboration, and evidence-based decision-making. They work in various capacities, including as bedside clinicians, as nursing directors, and in administrative roles, using their expertise to address complex policy issues, implement practice improvements, and foster interdisciplinary teamwork [9]. Their role extends from direct patient care to large-scale health policy transformation, positioning them as key drivers of sustainable healthcare reform.

Despite their importance, IL nurses lack clearly defined competencies [10]. Establishing these competencies is crucial to preparing them for their roles and ensuring they contribute effectively to healthcare transformation [9]. A structured competency framework would empower current IL nurses while guiding nursing education to equip future generations with the skills needed to navigate modern healthcare challenges. Addressing this gap would allow healthcare systems to maximize the potential of IL nurses, fostering evidence-based, sustainable improvements in patient care and policy development.

### Study Aim

This study develops a framework for IL nurses, identifying the core competencies needed to drive evidence-based policy transformation within healthcare systems.

## 2. Methods

This study utilized focus group interviews to gain insight into the key competencies essential for leading the implementation of evidence-based interventions and driving policy changes. These insights were crucial for informing the development of the Implementation Science Competence for Policy Transformation (ISCPT) framework, which aims to equip healthcare providers with the tools and knowledge necessary for effective policy transformation and sustainable change in clinical practice.

### 2.1. Participants

The study involved 12 IL nurses who participated in a PEACE-based workshop to enhance their competencies in leading EBP initiatives. All participants were responsible for overseeing the implementation of EBP initiatives within their respective healthcare settings. The nurses had varying levels of professional experience, ranging from 2 to 20 years, and represented a diverse mix of qualifications, including Master of Science in Nursing (MSN), Bachelor of Science in Nursing (BSN), and diploma degrees. The group consisted of 10 female nurses and 2 male nurses, holding various professional roles such as quality nurses, staff nurses, clinical instructors, and nursing administrators. Their experiences provided valuable insights into the challenges and success factors in implementing EBP within clinical practice.

### 2.2. Setting

The study was conducted under the EQUIP (Evidence-based Quality Improvement Project) initiative at a children’s hospital in Taif, Saudi Arabia, within the Taif Health Cluster. Nurses were trained in implementing EBP using the PEACE model, focusing on transforming healthcare policies and practices through structured training and capacity-building initiatives. EQUIP is closely aligned with Saudi Arabia’s Healthcare Transformation Program (HTP), a national initiative under Vision 2030, which mandates the integration of EBP into clinical and policy decisions across all health clusters. As part of this national transformation, the Taif Health Cluster is required to standardize care, enhance patient safety, and improve healthcare efficiency by ensuring that policies are guided by scientific evidence and best practices.

### 2.3. EQUIP Initiative

The EQUIP project served as the foundational initiative for developing the ISCPT framework. Established in 2023 through an academic–clinical partnership between Professor Al-Moteri (researcher) and Registered Nurse Aljuaid (clinician), this multi-phase project aimed to empower healthcare providers—particularly nurses—with EBP competencies to lead policy transformation and systemic change. EQUIP combines IS principles with a collaborative stakeholder engagement model, uniting clinicians, researchers, and policymakers to advance nursing practices and institutionalize sustainable improvements.

### 2.4. Data Collection

This study conducted a secondary analysis of qualitative data (SDA) from focus group interviews originally collected, recorded, and transcribed for the EQUIP project. SDA allows researchers to re-examine existing data to address new research questions beyond the original study’s scope [11]. While the parent study examined nurses’ experiences with EBP implementation, this analysis focuses on competencies essential for policy transformation. The dataset consists of focus group interviews conducted after nurses participated in a PEACE-based workshop [12]. The workshop addressed real-world clinical challenges, particularly low success rates in peripheral intravenous catheter (PIVC) insertion, identified through institutional quality reports. The interviews provided insights into systemic and role-specific factors affecting IL nurses’ ability to drive change.

Some authors of this study were participants in the original interviews, contributing first-hand insights into the policy transformation process in nursing practice. The dual role of researchers as participants was also acknowledged as an established qualitative research approach, which enhances contextual understanding and depth of analysis [13]. Researchers who also serve as participants gain unique insights into the study context, fostering greater reflexivity and a deeper appreciation of the lived experiences they examine [13].

### 2.5. Data Analysis

The transcribed interview data were re-analyzed using an inductive thematic analysis [14] structured approach. The analysis followed a two-phase process:

- Familiarization and Initial Coding—Researchers immersed themselves in the transcripts, identifying preliminary codes related to IL nurses’ competencies, challenges, and policy transformation strategies.
- Theme Identification and Refinement—The research team grouped codes into broader themes and subthemes, refining them through iterative discussions.

To ensure accuracy, themes were cross-checked against raw data to confirm theoretical saturation, ensuring no new insights emerged.

### 2.6. Data Rigor

Maintaining rigor and credibility in SDA is essential for ensuring valid and reliable findings [11]. This study employed multiple strategies to enhance trustworthiness and minimize bias in re-analyzing qualitative data. To strengthen triangulation and analytical depth, independent thematic coding was conducted by two researchers—one from the original research team and one newly involved in the SDA process [11]. Inter-coder reliability was assessed using Cohen’s Kappa to measure agreement between coders and ensure consistency in data interpretation. Discrepancies were discussed in structured group meetings, with a third researcher consulted to resolve persistent differences.

To further ensure credibility and transparency, member checking was conducted with a subset of participants to validate whether the findings accurately reflected their experiences. Additionally, an audit trail documented coding decisions, thematic refinements, and researcher reflections, enhancing reproducibility. Cross-referencing themes with raw transcripts ensured that findings remained grounded in participant narratives, confirming theoretical saturation.

By integrating independent coding, triangulation, member checking, and audit trails, this study upholds high methodological standards in qualitative SDA, ensuring the findings contribute reliably and transparently to understanding IL nurses’ competencies in policy transformation [11]. A key strength of this SDA is its continuity with the parent study, conducted in early 2024, followed by this re-analysis at the end of 2024, ensuring the timely relevance of the findings.

## 3. Findings

The thematic analysis revealed three key competency areas essential for IL nurses in evidence-based policy transformation (Table 1):

### 3.1. ISCPT Framework

**Figure 1:**
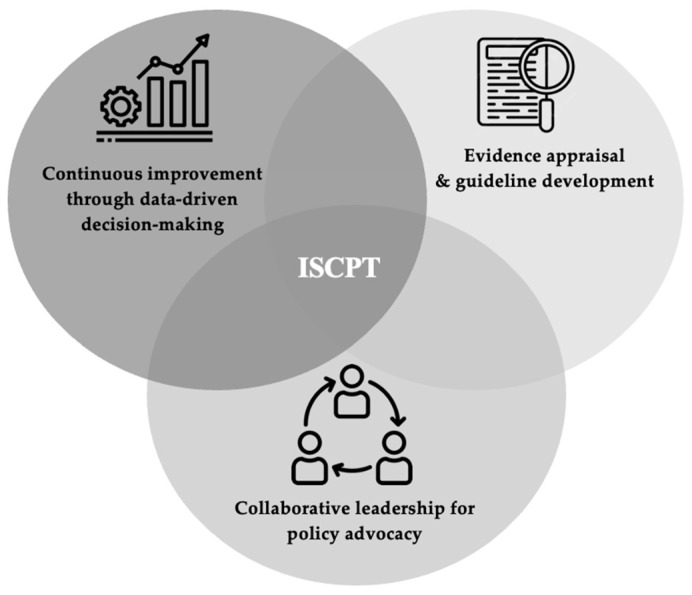
Implementation Science Competencies for Policy Transformation (ISCPT).

This study introduces the ISCPT framework, a nurse-driven model designed to operationalize evidence-based policy reforms in healthcare systems (Figure 1). Grounded in the experiences of IL nurses, the framework comprises three interrelated pillars that address gaps in policy translation, leadership, and sustainability (Table 1).

#### 3.1.1. Evidence Appraisal and Guideline Development

IL nurses critically appraise research quality and relevance (e.g., studies on PIVC insertion techniques) and synthesize findings into actionable, context-specific protocols. Participants emphasized tailoring guidelines to local challenges, such as reducing PIVC insertion failures by integrating audit data with best practices. For instance, one group described developing “*tailored guidelines that address PIVC failure rates and other clinical challenges*” (Group 3), ensuring interventions align with both evidence and institutional needs.

#### 3.1.2. Collaborative Leadership for Policy Advocacy

Nurses lead interdisciplinary teams to advocate for policy changes, bridging gaps between research and practice. This pillar emphasizes strategic communication with stakeholders (e.g., administrators, clinicians) to align priorities and address resistance. Participants highlighted leveraging PIVC success rate data to lobby for policy updates, as one group noted: “*Using audit data ensures informed decisions that drive measurable improvements*” (Group 1). Additionally, collaborative leadership fosters multidisciplinary engagement, with participants underscoring the importance of *“working with teams to ensure smooth policy implementation”* (Group 2).

#### 3.1.3. Continuous Improvement Through Data-Driven Decision-Making

Sustainable policy transformation relies on the iterative refinement of protocols and lifelong learning. IL nurses institutionalize changes by tracking outcomes (e.g., PIVC insertion metrics), identifying trends, and refining workflows. For example, one group described “*developing protocols to reduce failures and complications, then refining them through outcome tracking*” (Group 1). The framework also prioritizes continuous professional development (CPD), with nurses emphasizing the need for “*regular workshops to stay updated with evidence*” (Group 2), ensuring competencies evolve alongside emerging research.

## 4. Discussion

The findings of this study contribute to the development of the ISCPT framework, which addresses critical gaps in preparing nurses to lead evidence-based policy changes. By synthesizing insights from IL nurses’ experiences, the ISCPT framework outlines actionable competencies in evidence appraisal, leadership, and continuous improvement—skills essential for translating research into sustainable policy reforms.

### 4.1. Positioning the ISCPT Framework Within Existing IS Models

The ISCPT framework contributes to global implementation science by addressing two critical gaps: (1) the absence of role-specific competencies for nurses in policy transformation [15] and (2) the demand for scalable, sustainable models adaptable to diverse healthcare settings [16]. Unlike existing frameworks such as the Consolidated Framework for Implementation Research (CFIR) [17] and Promoting Action on Research Implementation in Health Services (PARIHS) [18], which primarily focus on identifying contextual barriers and facilitating evidence translation, ISCPT extends their applicability by equipping nurses with practical skills to overcome barriers. While CFIR highlights determinants influencing implementation outcomes (e.g., institutional inner/outer settings), ISCPT operationalizes these insights into structured competencies, such as evidence-informed advocacy and collaborative leadership, enabling nurses to mitigate barriers and drive systemic change.

The Knowledge-to-Action (KTA) framework [19] informs ISCPT’s approach to translating evidence into practice through knowledge creation, adaptation, and sustainability. However, KTA does not define competencies for policy transformation, limiting its utility in operationalizing evidence-based decisions. ISCPT addresses this by integrating nurse-specific competencies, such as evidence appraisal, guideline development, and continuous quality improvement (CQI), to execute KTA’s “action cycle” effectively. For example, ISCPT aligns with KTA’s “adapting knowledge to local context” phase by emphasizing tailored solutions to challenges like PIVC insertion failures. ISCPT extends KTA by embedding real-time data utilization, ensuring nurses refine initiatives dynamically, particularly in resource-limited settings.

### 4.2. ISCPT’s Unique Contribution

Compared to other models, ISCPT uniquely integrates nurse-led policy transformation as a core pillar. While CFIR and PARIHS focus on determinants or theoretical facilitation, ISCPT provides concrete guidance for nurses to institutionalize change through leadership, stakeholder engagement, and policy advocacy. It bridges the gap between theory and practice by embedding competencies that position nurses as decision-makers in implementation and policy reform. For instance, ISCPT’s emphasis on interdisciplinary collaboration and continuous professional development (CPD) ensures evidence translation evolves through iterative evaluation.

By incorporating leadership, CPD, and policy advocacy, ISCPT enhances traditional implementation science models, offering a scalable framework adaptable to diverse settings. Its focus on actionable skills, sustainability, and frontline nurse empowerment ensures policy reforms are contextually relevant and enduring. Table 2 summarizes these comparative advantages, underscoring ISCPT’s role in advancing implementation science from theory to action.

### 4.3. The Role of the PEACE Framework and the ISCPT Competency Model

The PEACE framework provides a structured methodology for implementing EBP, guiding nurses through key phases such as problem identification, evidence appraisal, and practice evaluation [7]. While both PEACE and ISCPT can serve as IS strategies, PEACE outlines the process, whereas the ISCPT framework defines the core competencies—such as collaborative leadership and data-driven decision-making—needed to execute these steps effectively [12]. ISCPT goes beyond PEACE by systematizing competency assessment and professional development, ensuring that training initiatives translate into measurable outcomes. By aligning competency development with implementation milestones, ISCPT enables institutions to rigorously evaluate the impact of PEACE-based workshops and structured training, positioning it as a methodological advancement rather than a repackaging of existing concepts.

### 4.4. Global Relevance and Policy Implications

The ISCPT framework aligns with global healthcare priorities, such as the WHO’s Strategic Directions for Nursing and Midwifery, which advocates for nurses to lead health system innovations. By institutionalizing competencies like data-driven decision-making [20] and interdisciplinary collaboration [21], ISCPT empowers nurses to advocate for policies that address disparities in care delivery—a critical need in low-resource settings [22]. For example, participants’ emphasis on refining PIVC protocols using audit data [12] demonstrates how ISCPT’s competencies can directly improve clinical outcomes while informing national policy agendas.

### 4.5. Strengthening Nursing Education and Practice

Structured CPD programs are critical for enhancing IL nurses’ competencies. Hospitals should prioritize systematic CPD over ad hoc training, including postgraduate programs in implementation science or EBP leadership, developed with universities to offer specialized modules on policy advocacy, data-driven decision-making, and interdisciplinary collaboration [3]. These programs should be paired with regular competency assessments—such as annual self-reviews and clinical audits—to evaluate nurses’ ability to translate evidence into policy. Biannual seminars and mentorship networks can address implementation barriers and foster leadership skills, while inter-hospital knowledge-sharing platforms enable collaborative learning and best-practice exchange. The ISCPT framework elevates CPD by embedding policy advocacy and implementation science competencies into nursing education. Traditional curricula often neglect these areas, leaving nurses ill-equipped for systemic reforms [23,24]. Integrating ISCPT through case studies [25], simulations [26], and leadership workshops [27] equips nurses with evidence appraisal and policy communication skills, essential for overcoming institutional resistance [28]. Regulatory bodies can further reinforce this by mandating periodic CPD certification, ensuring IL nurses stay aligned with evolving healthcare policies and implementation science advancements. Collectively, these strategies—postgraduate training, competency assessments, mentorship, and curricular integration—position evidence-based policymaking as a core nursing responsibility. They create a sustainable framework for lifelong learning, ensuring implementation science becomes integral to nursing practice and policy leadership.

### 4.6. Study Limitations and Future Directions

This study has several limitations that may affect the generalizability of its findings. The small sample size and single-site setting in Saudi Arabia may limit its applicability to healthcare systems with different institutional structures, resource levels, and nursing roles. While the 12 IL nurses provided valuable qualitative insights, future research should include larger, more diverse samples across multiple settings to improve the framework’s applicability. Exploring the adaptability of ISCPT in diverse cultural and resource contexts would further enhance the understanding of its effectiveness. Additionally, mixed-methods longitudinal studies could assess the long-term impact of ISCPT-driven policy changes on patient outcomes over time.

Another limitation is that all IL nurses in this study had previously participated in the PEACE-based workshop, which may have influenced their perceptions of ISCPT. Additionally, some authors of this study were also participants in the original interviews, meaning their dual role as both researchers and participants could have introduced unintended bias in data interpretation. While this insider perspective provided valuable contextual depth, it also required careful reflexivity and analytical rigor to minimize subjectivity. Future studies should consider including IL nurses who have not undergone PEACE training to provide comparative insights and further validate the framework’s applicability in different training contexts. Expanding research across diverse healthcare settings with varied policy environments would also help assess the scalability and adaptability of ISCPT.

### 4.7. Study Implication and Practical Application

The ISCPT framework enhances hospital systems by equipping nurses with skills to drive evidence-based policy reform, fostering cultures where nurses actively contribute to decision-making. By integrating ISCPT, hospitals can standardize care delivery, reduce practice variations, and ensure protocols align with the latest evidence—improving patient safety, reducing errors, and enhancing care quality [29,30]. Nurses trained in ISCPT systematically assess research, develop contextually relevant policies, and bridge gaps between frontline staff and administrators, ensuring policies reflect both clinical realities and strategic priorities. Additionally, ISCPT’s emphasis on data-driven decision-making enables hospitals to optimize workflows, address bottlenecks [31], and allocate resources efficiently, strengthening compliance with best practices.

Importantly, ISCPT is designed for adaptability across diverse settings, including smaller or resource-limited hospitals. In such environments, low-cost strategies like mentorship programs, peer collaboration, and simplified competency assessments can replace structured training, ensuring accessibility. The framework also allows for cultural adaptation, aligning competencies with local policies, workforce dynamics, and patient needs. This flexibility ensures ISCPT remains scalable, empowering institutions to advance evidence-based practice and policy leadership regardless of infrastructure constraints.

### 4.8. Barriers to Effective ISCPT Implementation

Despite its promise, integrating ISCPT into hospital systems presents several challenges. One major barrier is institutional resistance to nurse-led policy initiatives, which can limit their ability to drive change. Additionally, the lack of training in IS and limited data infrastructure in some settings hinder the successful adoption of ISCPT. In clinical practice, nurses often face time constraints, competing priorities, and inadequate support structures, making translating ISCPT principles into actionable change difficult. Without structured implementation strategies and leadership backing, sustaining evidence-based improvements becomes challenging.

Beyond clinical practice, integrating ISCPT into nursing education requires curriculum adaptation to include IS principles, policy advocacy skills, and data-driven decision-making. These areas are often underrepresented in traditional nursing programs, leaving nurses without the necessary competencies to implement and sustain policy reforms effectively.

Addressing these barriers requires strong leadership support, structured education programs, and investment in data management tools. Faculty development initiatives, interdisciplinary training programs, and mentorship opportunities could help equip nurses with the necessary skills to apply ISCPT effectively in real-world settings.

## 5. Conclusions

This study identifies the key competencies of IL nurses in driving evidence-based policy transformations and improving patient care, as outlined in the ISCPT framework. The ISCPT framework redefines nurses’ roles in implementation science by providing a structured, competency-based approach to policy transformation. By integrating lessons from global models like CFIR, PARIHS, and KTA—while addressing their sustainability and role-specific guidance gaps—ISCPT positions nurses as indispensable agents of health system change. For healthcare institutions, adopting this framework means investing not only in better policies but also in the nurses who turn evidence into action.

## Figures and Tables

**Table 1:** Themes and subthemes.

Themes	Subthemes	Quotes
Theme 1: Evidence appraisal and guideline development	Evidence appraisal and clinical application	“…I prioritize selecting interventions for PIVC management based on the latest research, ensuring they minimize complications and improve insertion success rates…” [Group 1]
	Guidelines development	“…this process has inspired me to develop tailored guidelines that address PIVC failure rates and other specific clinical challenges…” [Group 3]
Theme 2: Collaborative leadership for policy advocacy	Evidence informed policy advocacy	“…exploring EBP (evidence-based practice) has motivated me to update PIVC management policies, ensuring they align with the latest research and enhance patient outcomes…” [Group 2] “…collaboration with administrators is essential to advocate for evidence-based policies that improve care delivery…” [Group 3]
	Leadership in change management	“…I am prepared to lead my team through challenges, address concerns, and ensure the long-term benefits of adopting new practices…” [Group 1]
	Collaborative leadership	“…working with multidisciplinary teams is key to addressing PIVC-related issues. By fostering collaboration, we can ensure smooth and sustainable policy implementation…” [Group 2]
Theme 3: Continuous improvement through data-driven decision-making	Data-driven decision-making	“…audit data helps us identify trends in PIVC-related challenges, refine training, and adjust protocols for better outcomes… using data ensures informed decisions that drive measurable improvements…” [Group 1]
	Continuous quality improvement	“…developing protocols to reduce PIVC insertion failures and complications is essential. Tracking outcomes allows us to refine these protocols for sustained improvement…” [Group 1]
	Continuous professional development	“…training has reinforced the need to stay updated with new evidence and integrate it into daily practice. Regular workshops will be key to sustaining progress…” [Group 2]

**Table 2:** Differentiation of the ISCPT framework from established implementation of science models.

Framework	Primary Focus	Key Constructs	ISCPT Contribution
CFIR [16]	Implementation determinants	Inner/outer settings, intervention characteristics	Adds nurse competencies for policy translation
PARIHS [17]	Evidence–context facilitation	Evidence strength, readiness	Translates “facilitation” into leadership skills
KTA [18]	Knowledge-to-action cycle	Knowledge creation, application	Focuses on nurses’ role in institutionalizing change
ISCPT	Nurse-led policy change	Evidence appraisal, CQI/CPD, leadership	Integrates policy advocacy and sustainability

## Data Availability

All data generated or analyzed during this study are included in this published article.

## Abbreviations

The following abbreviations are used in this manuscript:

EBP	Evidence-based practice
IS	Implementation science
IL nurse	Implementation lead nurses
CQI	Continuous quality improvement
CPD	Continuous professional development
